# The Prevalence of Cardiac Risk Factors in Men with Localized Prostate Cancer Undergoing Androgen Deprivation Therapy in British Columbia, Canada

**DOI:** 10.1155/2015/820403

**Published:** 2015-08-02

**Authors:** Margot K. Davis, Jennifer L. Rajala, Scott Tyldesley, Tom Pickles, Sean A. Virani

**Affiliations:** ^1^Division of Cardiology, University of British Columbia, Vancouver, BC, Canada V5Z 1M9; ^2^Division of Cardiology, Royal Jubilee Hospital, Victoria, BC, Canada V8R 1J8; ^3^Department of Radiation Oncology, British Columbia Cancer Agency, Vancouver, BC, Canada V5Z 4E6

## Abstract

*Background.* While androgen deprivation therapy (ADT) reduces the risk of prostate cancer-specific mortality in high-risk localized prostate cancer, it adversely affects cardiovascular (CV) risk factor profiles in treated men. *Methods.* We retrospectively reviewed the charts of 100 consecutive men with intermediate- or high-risk localized prostate cancer referred to the British Columbia Cancer Agency for ADT. Data on CV risk factors and disease were collected and Framingham risk scores were calculated. *Results*. The median age of the study cohort was 73 years. Established cardiovascular disease was present in 25% of patients. Among patients without established CV disease, calculated Framingham risk was high in 65%, intermediate in 33%, and low in 1%. Baseline hypertension was present in 58% of patients, dyslipidemia in 51%, and diabetes or impaired glucose tolerance in 24%. Hypertension was more prevalent in the study cohort than in an age- and sex-matched population sample (OR 1.74, *P* = 0.006); diabetes had a similar prevalence (OR 0.93, *P* = 0.8). *Conclusions.* Patients receiving ADT have a high prevalence of cardiovascular disease and risk factors and are more likely to be hypertensive than population controls. Low rates of CV risk screening suggest opportunities for improved primary and secondary prevention of CV disease in this population.

## 1. Introduction

Prostate cancer is the most common cancer diagnosed in men, affecting 1 in 7 men in North America [1, 2]. Androgen deprivation therapy (ADT) with gonadotropin releasing hormone (GnRH) agonists has been a mainstay of therapy for locally advanced and metastatic prostate cancer since the 1990s and is also increasingly used in the neoadjuvant setting prior to radiotherapy for early disease [3].

While ADT decreases the risk of prostate cancer-specific mortality in advanced prostate cancer, it is associated with a number of adverse metabolic effects. The risk of developing diabetes while on ADT increases by up to 44% [4, 5] as lean body mass is significantly reduced and replaced by increased fat mass [6, 7]. Several large population-based studies have indicated that men receiving ADT are at increased risk of fatal and nonfatal cardiovascular events [5, 8, 9]. Moreover, men with cardiovascular risk factors or previous cardiovascular events are at particularly high risk [9, 10].

No specific guidelines exist for the screening of patients with prostate cancer who have preexisting cardiovascular disease or who may be at risk for cardiovascular morbidity and mortality as a result of ADT. Studies have shown that cancer patients and survivors may be less likely to receive therapies directed at cardiovascular risk factor modification compared to other patients [11]. Underrecognition of risk factors and subsequent undertreatment may represent an important care gap in survivor populations. As such, appropriate treatment and modification of cardiovascular risk factors may minimize treatment related adverse effects. With this in mind, we investigated the burden of cardiovascular risk among patients receiving ADT for intermediate- and high-risk localized prostate cancer at our centre and described the measures taken to risk-stratify these patients prior to therapy.

## 2. Methods

### 2.1. Patient Population and Treatment

The study population included 100 consecutive men with intermediate- or high-risk prostate cancer who were referred to the British Columbia Cancer Agency (BCCA) between October 1, 2011, and October 31, 2012, and who were treated with combined ADT and radiotherapy with curative intent. Patients were included if they had been referred to an oncologist within 3 months of cancer diagnosis and if their treatment plan included curative intent radiotherapy and ≥6 months of ADT. Patients were excluded if they had metastatic prostate cancer. Radiotherapy protocols and choices of ADT regimens were at the discretion of treating physicians.

### 2.2. Data Collection

Data was collected by retrospective chart review. Data on patient demographics, past medical history, prior cardiac history, and medications were collected from the oncology chart. If a specific CV risk factor was not mentioned in the past medical history, the patient was presumed not to have that risk factor unless (1) the patient was on antihypertensive medications, in which case they were considered to have hypertension, or (2) the patient was on a statin medication or their lipid profile revealed an LDL ≥3.5 mmol/L or a non-HDL-C of ≥4.3 mmol/L, in which case they were considered to have dyslipidemia. Systolic blood pressure was recorded from the initial consult with the oncologist or from the anaesthesia before operative consult or other specialist consults if not available on the first visit. The electronic chart and the common provincial laboratory system were accessed to find any lipid profiles from the year prior to or concomitant with the start of ADT. Lastly, the presence of any CV investigations on the chart, either in the year prior to the initiation of ADT or in response to the start of ADT, as well as any referral to a cardiology or internal medicine service for cardiac assessment was recorded. The University of British Columbia Research Ethics Board approved the data collection protocol used in this study.

### 2.3. Risk Calculation

A Framingham risk score (FRS) [12] was calculated on all patients who did not have underlying coronary heart, cerebrovascular, or peripheral arterial disease (PAD) to estimate the 10-year risk of CV events. A modified Charlson Comorbidity Index [13] was calculated on all patients to estimate 10-year mortality risk. Prostate cancer diagnosis was not accorded any points in the modified Charlson score. If no blood pressure was recorded on the chart, a score of 0 was used for hypertension in the Framingham risk calculation. Because ADT is known to increase serum lipids, any lipid measurements taken after the start of ADT were not used in the Framingham risk calculation. If no lipid profile was available, a score of 0 was used for the HDL and total cholesterol values in calculating the FRS.

### 2.4. Comparison Cohort

The prevalence of two cardiovascular risk factors, hypertension and diabetes, was compared to values reported for males aged 65 years and over in British Columbia in the 2011-2012 Canadian Community Health Survey, administered by Statistics Canada [14]. These population prevalence values refer to the proportion of the population who reported that they had been diagnosed by a health professional as “having high blood pressure” or “having type 1 or type 2 diabetes,” respectively.

### 2.5. Statistical Analysis

Proportions of men in the prostate cancer cohort and comparison cohort with hypertension and diabetes were compared using Chi-square tests. *P* values <0.05 were considered significant. Statistical analysis was performed using Microsoft Excel for Mac Version 14.4.6 (Microsoft Corporation, Redmond, WA).

## 3. Results

Baseline demographics for the study cohort are displayed in Table 1. The median age of the cohort was 73 years (range 50–87 years). Thirty (30%) subjects had moderately differentiated cancer (Gleason score 5–7) and 70 (70%) had poorly differentiated disease (Gleason score 8–10). The median initial PSA value was 12 ng/mL (IQR 8.2, 21.1). No patient had any evidence of metastatic prostate cancer on either bone scan or staging CT scan. Most patients (82%) had a modified Charlson Comorbidity Index of 0.

### 3.1. Baseline Cardiovascular Risk

Previously documented coronary artery disease (CAD) was present in 17% of patients, with 9 of these patients having had a past percutaneous coronary intervention (PCI) and 5 patients having undergone coronary artery bypass grafting (CABG) surgery. A history of stroke was present in 7%, and 5% had a history of PAD. A total of 25 patients were excluded from the Framingham risk calculation due to previous history of CAD, PAD, or stroke. A history of cardiac arrhythmia was documented in 10% of patients, 5 of whom had atrial fibrillation. The presence of baseline cardiac risk factors was common in this cohort (Table 2), with 58% of patients having a history of hypertension, 51% having a history of dyslipidemia, and 17% of patients having a history of diabetes. Only 4% of patients had no cardiac risk factors at all. Complete data to calculate a Framingham risk score was present for only 17% of patients; 62% of those studied did not have a lipid profile in the year prior to starting ADT and 58% did not have a blood pressure recording on the chart. Despite amending the Framingham risk calculation with scores of 0 for the missing data, only 1 patient was in the low risk Framingham category. Most patients, 69%, were in the high Framingham risk category and 30% were calculated to be at moderate Framingham risk.

### 3.2. Cardiovascular Investigations

**Figure 1:**
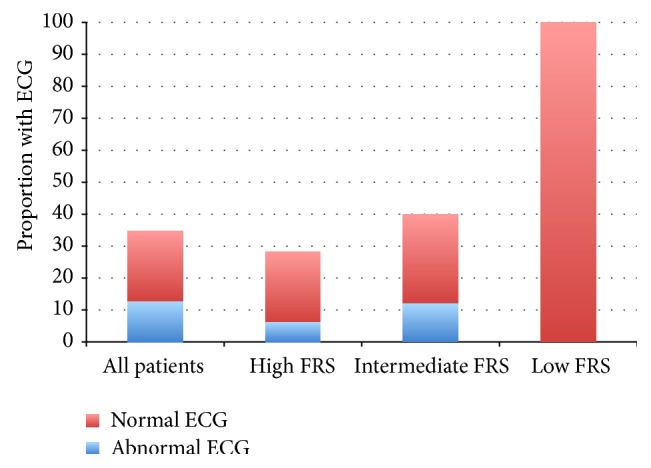
Proportions of patients referred for androgen deprivation therapy with normal and abnormal electrocardiograms available for review. ECG, electrocardiogram; FRS, Framingham risk score.

Only 35% of patients had an ECG present on the chart (Figure 1). 22 of 35 ECGs were classified as normal. The most common abnormalities noted on ECG were nonspecific ST segment changes (*n* = 5), followed by intraventricular conduction delays or bundle branch block (*n* = 3). Less common was evidence of a prior myocardial infarct (*n* = 2) or left ventricular hypertrophy (*n* = 2). Only 6% of the patients studied had further testing for cardiac ischemia with either exercise treadmill testing or myocardial perfusion imaging (Table 3). Documentation of a previous cardiology assessment was present for 6 patients, while 3 more patients were referred to a cardiology or internal medicine service for further work-up after oncology consult.

### 3.3. Comparison with Population Prevalence Values

In the 2011-2012 Canadian Community Health Survey, 44.3% (*n* = 139, 502) of men aged 65 years and over in British Columbia reported that they had been diagnosed with high blood pressure, and 18.0% (*n* = 56, 540) reported that they had been diagnosed with diabetes. Compared to the population sample, the odds ratio for having hypertension in our prostate cancer cohort was 1.74 (*P* = 0.006) and the odds ratio for having diabetes was 0.93 (*P* = 0.8).

## 4. Discussion

In this cross-sectional study of cardiovascular risk profiles among men referred for management of localized prostate cancer with ADT, we identified high prevalences of baseline cardiovascular risk factors and of cardiovascular disease. In addition to the 25% of men with established cardiovascular disease in our study population, at least 69% of subjects without established disease had a high FRS which is associated with a ≥20% 10-year risk of developing coronary heart disease, prior to initiation of ADT. By contrast, our cohort had a low prevalence of other comorbidities and a low expected mortality predicted by the Charlson Comorbidity Index.

We identified a higher prevalence of hypertension among our cohort than was reported among men aged 65 years and over in the same geographic area during the 2011-2012 Canadian Community Health Survey, suggesting that our prostate cancer population may be at higher risk of cardiovascular disease than members of the general population without prostate cancer, even after controlling for age and sex. The reasons for this association are not clear, but we can hypothesize that men with prostate cancer may have an increased burden of cardiovascular risk factors because many of these risk factors are also associated with risk of cancer. Indeed, previous studies have demonstrated increased prostate cancer risk among men with risk factors such as hypertension [15, 16], dyslipidemia [17], and the metabolic syndrome [15, 17, 18]. Increased prostate cancer risk has also been observed among men with established coronary artery disease [19]. Men with metabolic syndrome and hypertension are also at increased risk of biochemical recurrence following radical prostatectomy for prostate cancer [20–22]. Although the pathways by which the metabolic syndrome and its components increase cancer risk have not been fully elucidated, likely mechanisms involve insulin resistance, hyperinsulinemia, and elevated levels of insulin-like growth factor (IGF)-1, as well as increased levels of inflammation [17]. It is, therefore, somewhat surprising that diabetes, a state associated with insulin resistance, was not more prevalent in our cohort than in the general population. However, this finding was consistent with previous studies and it has been suggested that this may be because advanced diabetes is associated with low insulin levels due to pancreatic *β*-cell failure, resulting in a late protective effect [21].

ADT has been associated with worsening cardiovascular risk profiles and with major adverse cardiac events. Men taking ADT for prostate cancer have an increased risk of obesity, insulin resistance, and dyslipidemia [23]. Although a large meta-analysis of randomized controlled trials failed to demonstrate an increased risk of cardiovascular death among men randomized to ADT [24], several retrospective analyses of ADT in nontrial settings have found increased risks of fatal and nonfatal cardiovascular events, including myocardial infarction, stroke, and peripheral arterial disease [25, 26]. The use of this therapy in men with preexisting cardiovascular disease is associated with a particularly high risk of events [27–29]. In our cohort, this accounted for 25% of the study population.

An earlier study done at our centre found that men receiving ADT had a lower prevalence of cardiovascular disease and risk factors than men who did not receive ADT [30], suggesting that this therapy was being withheld from the highest risk patients, thereby identifying a significant treatment bias. Indeed, men with prostate cancer are twice as likely to die of cardiovascular disease as they are to die of prostate cancer [31]; cardiovascular disease is the leading cause of death in this population. This therefore represents a vulnerable population in whom effective interventions for the primary and secondary prevention of cardiovascular disease are likely to be of high yield. Patients receiving ADT, in whom the risk is expected to further increase with cancer treatment, warrant particular attention in order to minimize iatrogenic escalation of cardiovascular risk. Conversely, effective modification of cardiovascular risk factors to achieve guideline-recommended targets may result in a greater proportion of prostate cancer patients being deemed eligible for ADT, resulting in improved oncologic outcomes.

In our study cohort, large proportions of patients had not had blood pressure or lipid measurements done prior to initiation of ADT. Current European Association of Urology guidelines recommend that existing general population screening and treatment strategies should be applied to patients receiving ADT [32]. Canadian Cardiovascular Society guidelines recommend annual screening of lipids for individuals with calculated Framingham risks of ≥5% [33], and the Canadian Hypertension Education Program recommends that all adults should be screened for hypertension at all appropriate visits [34]. These guidelines have also addressed pharmacologic and nonpharmacologic strategies for risk factor modification. While management of these risk factors may fall outside the scope of typical oncology practice, cardiooncology clinics can offer multidisciplinary approaches to risk reduction. Cardiooncology clinics focus on prevention and management of cardiovascular disease in cancer patients and aim to remove cardiovascular disease as a barrier to effective cancer treatment [35]. An important aspect of this care is the management of cardiovascular risk factors. In addition, supervised exercise programs offer additional opportunities for risk reduction through physical activity and have been shown to improve cardiovascular risk profiles and cardiopulmonary fitness in prostate cancer patients [36–38].

Very few patients in our cohort had undergone any form of diagnostic testing for CAD. Although the resting ECG is an insensitive method of screening for silent CAD, abnormalities including Q-waves, ST segment depression, and bundle branch block have reported specificities of >95% for the prediction of cardiovascular mortality [39]. In our cohort, 37% of ECGs performed were abnormal, reflecting the higher prevalence of cardiovascular disease in our study cohort than in the population-based cohorts from which sensitivity and specificity analyses are derived [40]. Resting ECG may therefore be a useful screening test in this high-risk population.

Exercise stress testing is a far more sensitive method of screening for CAD than resting electrocardiography. Henriksson and Johansson reported that ischemic changes during an exercise stress test, in conjunction with abnormalities of blood lipids and hormone levels, were strongly predictive of cardiovascular events among men receiving estrogen therapy for prostate cancer [41]. To our knowledge, a similar study has not been performed in men receiving ADT, but the predictive value of exercise testing in the general population has been validated and is widely accepted [42]. Moreover, Wall and colleagues have reported that maximal exercise testing is feasible and safe in men receiving ADT for prostate cancer and have emphasized the importance of exercise testing among men receiving ADT in the context of additional risk factors who are embarking on an exercise program [43].

A strength of our study was the detailed chart review methodology, likely yielding a higher prevalence of comorbidities and higher rate of cardiac testing than would have been achievable with administrative data or with self-report. There are also limitations to our study. Our sample size was relatively small, potentially limiting the precision of our results. However, we included all patients meeting our inclusion criteria over an entire year and we believe that our sample was representative of the population seen at our centre. In addition, as ADT may be withheld from patients at the highest risk for cardiovascular complications, our results cannot be generalized to all prostate cancer patients. As discussed above, we have reason to believe that the broader prostate cancer population carries, if anything, a greater risk than our cohort [30]. For comparison purposes, we were only able to obtain local population data on the prevalence of hypertension and diabetes, and we were therefore unable to compare the prevalence of cardiovascular disease or dyslipidemia in our cohort to the general population. Our findings, however, were consistent with other epidemiologic studies that have identified associations between cardiovascular risk factors and prostate cancer risk, and the fact that we were able to illustrate the increased prevalence of even a single risk factor highlights the increased risk that may be present in this population. Finally, many of our subjects were missing the necessary data to accurately calculate an FRS. However, our use of zero scores for missing data points has resulted in a “minimal estimate” of risk, so we may be confident that we have not overestimated the risk of our cohort.

In conclusion, the results of the present study indicate that despite a low overall comorbidity burden, established cardiovascular disease and cardiovascular risk factors are common among men receiving ADT for intermediate- and high-risk localized prostate cancer. Certain risk factors are more common among prostate cancer patients referred for ADT than among population controls, possibly reflecting the fact that many cardiovascular risk factors are associated with increased prostate cancer risk and highlighting the particularly high-risk nature of this population. Moreover, our findings suggest that suboptimal risk stratification occurs in this population, and accordingly, suboptimal risk modification may result. As cardiovascular disease is a leading cause of death in men with prostate cancer and given that ADT is associated with increased risk of fatal and nonfatal cardiovascular events, this population is likely to benefit from aggressive primary and secondary prevention therapies. The findings of the present study identify an important care gap and an opportunity to improve survivorship care in this population. We propose that care processes to identify at-risk individuals, including standard cardiovascular risk factor assessment and modification or referral to a cardiologyoncology clinic where available, be broadly applied to this patient population to improve patient specific cardiac and cancer related outcomes.

## Figures and Tables

**Table 1:** Baseline characteristics of 100 men referred for androgen deprivation therapy.

Age at diagnosis	73 (50–87)
Vascular disease	25 (25%)
Coronary artery disease	17 (17%)
Percutaneous coronary intervention	9 (9%)
Coronary artery bypass surgery	5 (5%)
Stroke	7 (7%)
Peripheral arterial disease	5 (5%)
Atrial fibrillation	5 (5%)
Supraventricular tachycardia	1 (1%)
Arrhythmia, not specified	4 (4%)
Cardiac pacemaker	1 (1%)
Pericarditis	1 (1%)
Coronary vasospasm	1 (1%)
History of heart failure	1 (1%)
Framingham risk category	
High risk	49 (65%)^*^
Intermediate risk	25 (33%)^*^
Low risk	1 (1%)^*^
Gleason score	
Moderately differentiated (5–7)	30 (30%)
Poorly differentiated (8–10)	70 (70%)
Clinical stage	
T1	27 (27%)
T2	43 (43%)
T3	23 (23%)
T4	3 (3%)
X	4 (4%)
Initial PSA (ng/mL)	
<5	9 (9%)
5–10	29 (29%)
>10	62 (62%)
Updated Charlson Comorbidity Index	
0	82 (82%)
1	14 (14%)
≥2	4 (4%)
Hormonal treatment used	
Goserelin	72 (72%)
Leuprolide	25 (25%)
Degarelix	2 (2%)
Bicalutamide	92 (92%)
Buserelin	1 (1%)
Flutamide	1 (1%)

Expressed as median (range) or number (percentage).

^*^Expressed as percentage of patients without baseline history of vascular disease.

**Table 2:** Cardiac risk factors present in 100 men prior to receiving androgen deprivation therapy.

Risk factor	All patients (*n* = 100)	High FRS (*n* = 49)	Intermediate FRS (*n* = 25)	Low FRS (*n* = 1)
Hypertension	58 (58%)	28 (57%)	7 (28%)	0
Diabetes mellitus or IGT	22 (22%)	11 (22%)	6 (24%)	0
Cigarette smoking				
Never	46 (46%)	22 (45%)	14 (56%)	1 (1%)
Current	6 (6%)	3 (6%)	2 (8%)	0
Quit < 5 years ago	3 (3%)	1 (2%)	1 (4%)	0
Quit > 5 years ago	44 (44%)	23 (47%)	7 (28%)	0
Family history of CAD	10 (10%)	2 (4%)	0	0
Dyslipidemia	51 (51%)	19 (39%)	10 (40%)	0
Chronic kidney disease (GFR < 60 mL/min/1.73 m^2^)	10 (10%)	6 (12%)	1 (4%)	0
Medication class				
ASA	33 (33%)	12 (24%)	2 (8%)	0
Clopidogrel	1 (1%)	0	0	0
Warfarin	3 (3%)	3 (6%)	0	0
ACE inhibitor	32 (32%)	16 (33%)	2 (8%)	0
ARB	16 (16%)	8 (16%)	2 (8%)	0
Beta blocker	17 (17%)	7 (14%)	1 (4%)	0
Other antihypertensives	31 (31%)	15 (31%)	4 (16%)	0
Statin	40 (40%)	13 (27%)	6 (24%)	0
Nitroglycerin	1 (1%)	0	0	0
Insulin	4 (4%)	3 (6%)	0	0

ACE: angiotensin converting enzyme; ARB: angiotensin receptor blocker; ASA: acetylsalicylic acid; CAD: coronary artery disease; FRS: Framingham risk score; GFR: glomerular filtration rate; IGT: impaired glucose tolerance.

**Table 3:** Cardiac evaluations performed in men with prostate cancer referred for androgen deprivation therapy.

Investigation	All patients (*n* = 100)	High FRS (*n* = 49)	Intermediate FRS (*n* = 25)	Low FRS (*n* = 1)
Echocardiogram	3	1 (2%)	1 (4%)	0
Exercise treadmill test	3	1	1	0
Positive for ischemia	1	0	1^†^	0
Negative for ischemia	2	1	0	0
Myocardial perfusion imaging	2	0	0	0
Positive for ischemia	1	0	0	0
Negative for ischemia	1	0	0	0
Stress echocardiogram	1	0	1	0
Positive for ischemia	0	0	0	0
Negative for ischemia	1	0	1^†^	0
Holter monitor	1	1	0	0
Normal	1	1	0	0
Abnormal	0	0	0	0

FRS: Framingham risk score.

^†^One patient had evidence of ischemia on exercise treadmill testing and was subsequently referred for stress echocardiogram which showed no ischemia.
